# Human Permanent Ectoparasites; Recent Advances on Biology and Clinical Significance of *Demodex* Mites: Narrative Review Article

**Published:** 2017

**Authors:** Dorota LITWIN, WenChieh CHEN, Ewa DZIKA, Joanna KORYCIŃSKA

**Affiliations:** 1. Dept. of Medical Biology, Faculty of Medical Sciences, University of Warmia and Mazury, Olsztyn, Poland; 2. Dept. of Dermatology and Allergy, Ludwig-Maximilian-University of Munich, Munich, Germany; 3. Women’s Health Center, Far Eastern Memorial Hospital, New Taipei, Taiwan

**Keywords:** *Demodex folliculorum*, *Demodex brevis*, Demodicosis, Ectoparasites

## Abstract

**Background:**

*Demodex* is a genus of mites living predominantly in mammalian pilosebaceous units. They are commonly detected in the skin of face, with increasing numbers in inflammatory lesions. Causation between *Demodex* mites and inflammatory diseases, such as rosacea, blepharitis, perioral and seborrhoeic dermatitis or chalazion, is controversially discussed. Clinical observations indicate a primary form of human *Demodex* infection. The aim of this review was to highlight the biological aspects of *Demodex* infestation and point out directions for the future research.

**Methods:**

We conducted a broad review based on the electronic database sources such as MEDLINE, PubMed and Scopus with regard to the characteristics of the *Demodex* species, methods of examination and worldwide epidemiology, molecular studies and its role in the complex human ecosystem.

**Results:**

*Demodex* mites are organisms with a worldwide importance as they act in indicating several dermatoses, under certain conditions. However, correlations between *Demodex* and other parasites or microorganisms occupying one host, as well as interactions between these arachnids and its symbiotic bacteria should be considered. There are few methods of human mites’ examination depending on purpose of the study. Nevertheless, paying attention must be needed as polymorphism of *Demodex* species has been reported.

**Conclusion:**

Overall, the present review will focus on different aspects of *Demodex* mites’ biology and significance of these arachnids in human’s health.

## Introduction

There are three main groups of parasites that can cause disease in humans: protozoans, helminths and ectoparasites. Parasitic mites of humans include chiggers (i.e. *Trombicula autumnalis*)*,* human scabies (*Sarcoptes scabiei var. hominis*) and *Demodex* mites. Among them, only *Demodex* mites are permanent ectoparasites of human and other mammalian pilosebaceous unit. A total of 140 species or subspecies have been identified worldwide in 11 orders of mammals including humans (1). Human *Demodex* have been found in nearly all age and racial groups (2–4).

There are two species of parasitize humans: *D. folliculorum,* and *D. brevis*. The first usually lives in the follicular infundibulum, while *D. brevis* is located in sebaceous and Meibomian glands (5). They infect mainly skin of the face and scalp, although both species were originally found in the ear canal (6). *D. folliculorum* often occupies follicles of the eyelashes (7–9). Identification of *D. folliculorum* in the nipple discharge (10) or in biopsy specimens of nevi (11) and skin cancers (12) has been reported.

Every human being carries a colony of 1000 to 2000 *Demodex* mites (13). Although *Demodex* mites are considered to be highly host species-specific, cross-infections between humans and animals have been documented (14, 15). However, the reliability of these rare case reports remains to be verified, particularly because of the polymorphism reported in *D. canis* and *D. folliculorum* (16,17). Many studies have shown higher density of the parasites in diseased inflammatory skin than in normal skin, but whether it is the cause or result of the inflammation remains unclear (18–20). When compared to well-known skin microorganisms inhabiting the human microbiome, such as *Propionibacterium acnes, Staphylococcus epidermidis* and *Malassezia*, *Demodex* mites possess a much higher hierarchy in the timeline of life evolution.

Therefore, it is really important to know better the biology of *Demodex* mites in order to understand the complex relationship between these mites and humans and most of all, to apply the right treatment when needed.

## Methods

We conducted a review based on the database sources such as MEDLINE, PubMed, and Scopus. Keyword used for searching all valuable information were: *Demodex*, Demodicidae, human mites and its combinations with all words regarding to biological characteristic of the species and interactions among one host organism, epidemiology and methods of examination or molecular studies. The search included the articles published up to the 2015. No restrictions were placed on study design or language of publication.

## Results

### Classification, morphology and life cycle

Mites are small arthropods belonging to the subclass Acari. They live in an enormous number of habitats; often spend their entire lives as parasites. *Demodex* mites belong to the superorder Acariformes, order Trombidiformes, suborder Prostigmata, superfamily Cheyletoidea, family Demodicidae and genus *Demodex* (1). In humans, parasitize two species: *D. folliculorum* and *D. brevis*. Lately, the phenotypic differentiation among the former has been found (16). Mites of the family Demodicidae are tiny organisms (usually 0, 2–0, 4 mm). Most of them have vermiform elongation of the body, which consists of three main parts: gnathosoma, podosoma and opisthosoma. Four pairs of legs are spaced along the podosoma, each with a pair of claws (21). The mouthparts include a round oral opening with a spindle hypostome and stylet-like chelicerae (21) as they feed on the contents of sebum and epithelial cells. The limitations of these mites to the dimensions of hair follicles have resulted in a reduction of organ systems. They do not have a tracheal system and their digestive system is highly modified, consisting of: the chelicerae and a poorly formed midgets lumen with no hindgut and anus (22). The latter helps to avoid triggering an immune response from the host, but there are issues with waste excretion that consequently leads to a time-limited life.

*Demodex* spends its entire life cycle (14–18 d) on the host (8). The reproduction is sexual, involving adult males mating with adult females. Nymphs are probably ignored, as they are not sexually matured. The male genital orifice is placed dorsal, between a second pair of legs, while the vulva extends ventrally at the level of the fourth pair of legs (23). Adults copulate in the opening of the hair follicle (8). There is no data on mating behavior, but we can suppose it goes similarly to other mites: male and female face in opposite directions, the penis is inserted into the female opening and sperm are transferred to the female (24). Copulation may take up to 48h in house-dust mites, during which time the female is mobile (24). The eggs of *Demodex* mites are lying inside the hair follicles or sebaceous glands. The subsequent stages are larva, protonymph, deutonymph and adult (25).

### *Demodex* culture model

The only way to obtain *Demodex* mites is collect them from human beings, thus setting up culture model would greatly increase possibilities of research. Their maintenance in vitro has not been achieved yet, because of their ease of dying. However, the effect of temperature and medium on the viability in vitro of *Demodex* mites has been studied (26). The activity of the mites is related to the photoperiod and the temperature (27). *Demodex* mites are photonegative (27). The optimal temperature in vitro for both: *D. folliculorum* and *D. brevis* is about 16–22 °C while in the 36–37 °C (so in the temperature of human body) they lived shortest (28). Survival of both species was longest on human serum and 1640/seroculture solution (28).

In developing ex vivo culture model of *Demodex* mites’ artificial skin can be helpful. The model of human skin tissue has been developed yet but the presence of hair follicles and blood vessels are crucial for cultivating human mites. Preliminary studies of German scientists on the combined skin are promising (29).

### Molecular studies

The presence of hard chitinous exoskeleton makes *Demodex* mites difficult to study at the molecular level. Therefore, searching for their genotype has only started recently (17, 30). The first partial DNA sequence of *D. canis* chitin synthase (CHS) was submitted to Gen-Bank (No. AB080667). The CHS gene fragments of *D. canis* and *D. brevis* were cloned and sequenced with results showing similarities at a level of 99.1%–99.4% between these two species (31). In the same year, genomic DNA extraction from individual *Demodex* mites was carried out successfully (1).

The genetic relationship between *D. folliculorum* and *D. canis* is closer than that between *D. folliculorum* and *D. brevis* (32). The prediction of the secondary structure for the complete rDNA sequence of *D. folliculorum* was focused (33). In the effort to identify inter- and intraspecies variation, the cytochrome oxidase I (*cox1*) gene region is a useful tool in discriminating between populations such as those of *D. folliculorum* (17). Intraspecies variations based on *cox1* and mitochondrial 16S rDNA (16S mtDNA) was evaluated (1, 34). There were no geographical differences existing among *Demodex* isolates from Spain and China. However, differences in the *cox1* gene were observed between populations of *D. folliculorum* from facial skin and eyelids, caused by variations in the local environment (34).

*Cox1* gene encodes protein that is the component of the respiratory chain, which catalyzes the reduction of oxygen to water. That gene has more rapid evolution rates than 16S mtDNA, therefore, is more useful for the phylogenetic analysis of closely related species, subspecies and different geographic populations (34). Although 16S mtDNA was not suitable for intraspecies determination of *Demodex* (1), it seems to be applicable for phylogenetic relationship analysis in low taxa (30). Similarly, 18S rDNA was used for interfamily identification in Cheyletoidea (1).

Lately, the molecular identification of four phenotypes of human *Demodex* mites demonstrated long- and short-bodied *D. folliculorum* with finger-like terminus and *D. brevis* with finger- or cone-like terminus (35). The molecular data of *D. brevis* with finger-like terminus, morphologically classified as *D. brevis*, was molecularly identified to be *D. folliculorum* and it might be a morphological variant of *D. folliculorum* (35). Therefore, there is polymorphism among *D. folliculorum* that can be associate with the skin type of hosts, parasitic site and the source of nutrition (16). Similar findings have been published in the study of *Demodex* in dogs, where *D. canis*, *D. injai* and *D. cornei* previously considered as three distinct species, from genetic distance and divergence data are regarded as polymorphism of the same species (17).

Therefore, attention must be paid on *Demodex* species indicating.

### Methods of examination

There are no standard methods for the examination of human *Demodex* mites. To collect mites for further research, the cellophane tape method (CTP) (36), squeezing method (5), or skin scrapings can be used. CTP seems to be more effective with a positive rate at 91%, whereas squeezing gives a 34% positive diagnosis (37). Standardized Skin Surface Biopsy (SSSB) is the most commonly used method for comparing densities of mites between patients with dermatoses and healthy controls (38–40). The method consists of placing a drop of cyanoacrylic adhesive on a microscope slide, applying the adhesive-bearing surface of the slide to the skin, and removing it gently after it has been allowed to dry (about 1min). Initially, a standard surface area of 1cm^2^ is drawn on the slide (41). SSSB is a noninvasive sampling method by which it is possible to collect a superficial part of the horny layer and the contents of the pilosebaceous follicle (41). As compared to direct microscopic examination of fresh secretions from sebaceous glands, SSSB and has a higher sensitivity to measure the density of Demodex mites (42). More than five mites per cm^2^ are assumed a positive diagnosis of demodicosis (38, 42). The validity of this optimal threshold is rather artificial and weakly evidence-based (4). Skin punch biopsy for the detection of human mites is less frequently used because of its invasive character. For the study of eyelid involvement, a few eyelashes from each eye are epilated and placed on the slide in Hoyer’s liquid for microscopic examination (2, 3). Dermoscopy, reflectance confocal microscopy (RCM) and confocal laser scanning microscopy (CLSM) have been recently shown to measure *Demodex* mite density (43–45). They are non-invasive imaging methods with the advantage of visualizing in vivo structures with low (dermoscopy) or high resolution (RCM and CLSM). They enable detect and quantify *Demodex* mites per follicle or per evaluated area (44). Biological examination of such mites cannot be carried out as they are only partially seen although for diagnostic approaches they seem to be effective methods but expensive ones.

### Epidemiology

Human mites are ubiquitous and present in all races (2, 37). In general, most humans are infested by *D. folliculorum* but *D. brevis* is often found in the same host (46–48). The difference between the number of two mite species was pronounced the most in eyelid hair follicles, *D. brevis* recorded rarely (2,3). The total infestation rate in different study groups range usually from 17 to 72% in healthy humans, reaching as high as 100% in people over 96 yr old (49). Reports of less than 100% prevalence are probably due to the sampling methods used (13).

### Age

The incidence of infestation increases with age (2, 3, 50). High intensity of infection by *Demodex spp.* at the level of 44%–86% was noted in age groups: 17–25, 26–34 and over 35 yr (3). *Demodex sp.* occurs among: 13% of the people aged from 3 to 15 yr; 34% of the people aged from 19 to 25 yr; 69% of the people aged from 31 to 50 yr; 87% of the people aged from 51 to 70 yr and 95% of the people aged from 71 to 96 yr (51). Only 11% of healthy children fewer than 10 yr old are infected (2), although there are some reports of numerous *D. folliculorum* being found in immunocompetent children aged between 10 months and 5 yr (52). In Brazil, the prevalence was high in all age groups (46). That may be due to the humid-subtropical climate, but it is hard to confirm this assumption as many other factors influence the prevalence of human mites.

### Gender and skin type

The gender impact on the prevalence rate of mites of the genus *Demodex* is controversially discussed, with male predominance (47–49), female predominance (40, 50) to no difference (2,37). No difference was showed in the mite density between pregnant women and age-matched non-pregnant controls (53). In Chinese population, people with oily or mixed skin showed a higher prevalence of infestation than those with dry or neutral skin (37,50), where persons with oily cutis had increased amounts of *D. folliculorum* on the skin surface than those with dry cutis (54).

### Immune status

Studies indicate increased number of *D. folliculorum* in immunocompromised patients: with end stage chronic renal failure, diabetes, Behçet’s disease, urological cancers and eyelid basal cell carcinomas (55–59). Among epidermal neoplasms on the face, the highest infestation rate of *D. folliculorum* was in cases of nasal epidermal neoplasm compared with other locations (60). Children malnutrition indicated a much higher prevalence of mites (25%) than control groups (1.6%) (61). However, the prevalence of human mites in patients with chronic kidney deficiency and rheumatoid arthritis was similar when compared to the control groups (62, 63). There was a positive correlation between human demodicosis and certain haplotypes of HLA (Human Leucocyte Antigen) class I, which involve in immune reactions. HLA A2 was revealed the resistance marker for the development of demodicosis (64). It remains to be determined which kind of cellular immunity may foster mites’ proliferation.

### Skin diseases

The pathogenic role of human *Demodex* mites in certain inflammatory skin disorders is debating. A higher prevalence of mites has been observed in rosacea (19, 39), seborrhoeic dermatitis (20), perioral dermatitis (7), blepharitis (65, 66) and chalazion (67, 68). Recently human primary demodicosis has been recognized as a primary disease sui generis and a clinical classification has been proposed (4). A secondary form of human demodicosis is mainly associated with systemic or local immunosuppression (4).

### Double-faced Demodex as a part of complex human ecosystem

Demodectic mange in many animals (e.g. dogs) is a potentially lethal condition. It is caused by an abnormal proliferation of the normal mite population and it is commonly complicated with a secondary bacterial folliculitis and furunculosis (69). Several pathogenic mechanisms have been proposed by which *Demodex* mites may cause or aggravate skin conditions. Mechanically, they can block pilosebaceous ducts causing epithelial hyperplasia and hyperkeratinization (8). The enzymatic activity of mites causes damage to the glandular and epithelial cells lining the hair follicles, leading to the induction of inflammation (38, 39). Antigens of the parasite can also raise an immunological reaction (70). Mites can give rise to an inflammation cascade rather than causing direct damage to tissue (71). Moreover, *Demodex* mites contain lipase, supposed to be able to aggravate skin conditions by transforming sebum into certain components, which are clearly cytotoxic, and irritants (72). *D. folliculorum* might act as a vector for *Bacillus oleronius*, which is most likely a co-pathogen in the development of blepharitis (9). Nevertheless, in most cases of *Demodex* infestation it is asymptomatic. Thus, the type of symbiosis between human and *Demodex* mites remains unclear. Human mites act like commensals and only their overgrowth may be the cause of the disease state. That overgrowth can be caused by imbalance between mite’s virulence factors and the host’s response (73). The situation becomes worse when hormonal abnormalities or chronic diseases in the host organism coexist. However, we need to see complexity of interactions among one organism, not only one host-one symbiont interactions. Correlations between parasites inhabiting one host should be of our interest as harbouring multiple parasite species by one human is rather ubiquitous in nature. Interactions between parasites may effect on disease severity, response to the drugs and many more, i.e. nematodes are well known for their immunomodulatory effects, in particular, their suppression of adaptive immune responses (74). Mites alone are also able to exert an immunosuppressive effect on the cats (75).

Another type of correlations is that among micro-inhabitants of the host as human skin surface forms a complex ecosystem, consisting of i.e. bacteria (e.g. *S. epidermidis*), yeasts (*Malassezia furfur*) or mites (*Demodex* mites). *Demodex* may have a synergistic relationship with bacteria from genus *Staphylococcus*. Bacterial antigens work with host antibodies to inhibit the host’s response and therefore favor the multiplication of both given organisms (76). *Malassezia pachydermatis*, which is capable of causing skin inflammation, is reported to multiply better in the presence of staphylococci, but no interaction between *Malassezia* and *Demodex* is known (76).

Moreover, arthropods have their own symbiotic bacteria in which relationships varied from beneficial to harmful. The most abundant bacterial endosymbiont among arthropods is *Wolbachia* (77). W*olbachia* are able to modify the host’s reproductive system to their own advantage that is why was considered only as a harmful (78). Although lately evidences of *Wolbachia* mutualisms in arthropods was found, like in the bedbug *Cimex lectularius*, where bacteria provide essential B vitamins or in the mosquito *Culex pipiens*, where protect its host against *Plasmodium*-induced mortality (77). *Wolbachia* directly interferes with viruses and other pathogens inside the arthropod host. This *direct effect* of *Wolbachia* can either impede or promote the pathogen’s replication and survival (79). However, *Wolbachia* was not found in *Demodex* mites yet (80). Although, bacterium *Bacillus oleronius*, found inside the mite and probably act there like a symbiont, is able to produce proteins causing skin inflammation (9).

### Treatment of demodicosis

There are several treatment options of demodicosis available, including oral and topical drugs, but none of them has 100% efficiency (81). Acaricides has been used (i.e. ivermectin) as well as antibacterial antibiotics (i.e. tetracycline), supporting hypothesis about contribution of endosymbiotic bacteria in the induction of demodicosis (81, 82). Metronidazole and ivermectin are antiparasitic drugs, which administered both orally show relatively high efficiency – 71.6% of patients showed complete remission in case of rosacea and blepharitis (82). In those, who were treated with ivermectin alone, 45% of patients were much-cured (82). Topical medicaments may be used as an addition to oral drugs– topical ivermectin displays antimicrobial, antiparasitic, antibacterial, and anti-inflammatory activities (83). Using of tea tree oil ointment also reduced number of *D. folliculorum* on the eyelids (84) and can be applied as an additional treatment option. The main issue is common recurrence of demodicosis. Combined therapy can be more efficient than monotherapy but in order to find the best way to cure demodicosis, in vitro culture of human mites needs to be established.

## Conclusion

*Demodex* mites are organisms with a high worldwide importance as they are ubiquitous in humans and they role in indicating several dermatoses is quite sure, at least in certain conditions. Differentiation of demodicosis on primary (symptoms are directly caused by excessive mite population) and secondary (initially linked to local or general immunosuppression, secondarily with the increased mites’ number) seems corresponding to the facts. Directions of future research are for sure: 1) ex vivo (or *in vitro*) culture of the mites and hence finding effective methods of demodicosis treatment, 2) find endosymbionts and specific enzymes in *Demodex* mites in order to determine other pathogenic mechanisms in which they can act, 3) study polymorphism among *D. folliculorum* – reclassification may be needed.
